# Tools for annotation and comparison of structural variation

**DOI:** 10.12688/f1000research.12516.1

**Published:** 2017-10-03

**Authors:** Fritz J. Sedlazeck, Andi Dhroso, Dale L. Bodian, Justin Paschall, Farrah Hermes, Justin M. Zook

**Affiliations:** 1 Human Genome Sequencing Center, Baylor College of Medicine, Houston, TX, USA; 2 Worcester Polytechnic Institute, Worcester, MA, USA; 3 Inova Translational Medicine Institute, Inova Health System, Falls Church, VA, USA; 4 University of California, Berkeley, Berkeley, CA, USA; 5 Virginia Commonwealth University, Richmond, VA, USA; 6 Genome-scale Measurements Group, National Institute of Standards and Technology, Gaithersburg, MD, USA

**Keywords:** structural variants, whole genome sequencing, bioinformatics, NGS, annotation

## Abstract

The impact of structural variants (SVs) on a variety of organisms and diseases like cancer has become increasingly evident. Methods for SV detection when studying genomic differences across cells, individuals or populations are being actively developed. Currently, just a few methods are available to compare different SVs callsets, and no specialized methods are available to annotate SVs that account for the unique characteristics of these variant types. Here, we introduce SURVIVOR_ant, a tool that compares types and breakpoints for candidate SVs from different callsets and enables fast comparison of SVs to genomic features such as genes and repetitive regions, as well as to previously established SV datasets such as from the 1000 Genomes Project. As proof of concept we compared 16 SV callsets generated by different SV calling methods on a single genome, the Genome in a Bottle sample HG002 (Ashkenazi son), and annotated the SVs with gene annotations, 1000 Genomes Project SV calls, and four different types of repetitive regions. Computation time to annotate 134,528 SVs with 33,954 of annotations was 22 seconds on a laptop.

## Introduction

The advent of high throughput sequencing (HTS) facilitates the investigation of genomic differences among and within organisms, populations, and even diseases such as cancer. While the identification of single nucleotide polymorphisms (SNPs) is currently well established, structural variant (SV) calling remains challenging and little is known about the sensitivity (correctly inferring SVs) and false discovery rate (FDR) (falsely inferring SVs) of structural variation detection ( Guan & Sung, 2016). Recent SV discovery methods, such as LUMPY ( Layer *et al.*, 2014) and PBHoney ( English *et al.*, 2014), focus on one callset per technology, but SV detection and call evaluation would benefit from comparison of the data from multiple technologies. However, many challenges exist in comparing and merging SV calls due to uncertainty in breakpoints, sequencing errors, and multiple possible representations of SVs in repetitive regions ( Wittler *et al*., 2015). In addition, Sudmant *et al.* ( Sudmant *et al.*, 2015) as well as Jeffares *et al.* ( Jeffares *et al.*, 2017) mention that the methods often lack sensitivity and suffer from an inestimable FDR. Jeffares *et al.* coped with this problem by merging SV calls generated by multiple callers to reduce the FDR, but this approach also slightly reduced sensitivity ( Jeffares *et al.*, 2017).

To enable comparison and evaluation of SV callsets generated by different algorithms, we developed methods to compare and annotate SV calls, represented in variant call format (VCF), with other SVs as well as other genomic features. Genomic features can include gene annotations, mappability tracks, and any feature that can be represented as a region in BED or GFF format. SNPs can also be used for annotation by representing them as regions of 1bp.

As a proof of concept, we apply these novel methods to the Genome in a Bottle (GiaB) data generated on the Ashkenazi son (NIST Reference Material 8391, aka HG002 and NA24385) to explore SV type and breakpoint concordance of SV calling algorithms. GiaB provides SV calls generated using five different technologies (including Illumina short read sequencing, Complete Genomics nanoball sequencing, Pacific Biosciences long read sequencing, 10X Genomics linked reads, and BioNano optical mapping) and 16 different SV calling algorithms on the same genome. We used SURVIVOR ( Jeffares *et al.*, 2017) to merge SV calls and our novel method (SURVIVOR_ant) to annotate and predict more precise breakpoints. All data sets (including merged SV and annotated SV) and methods used in this manuscript are available at https://github.com/NCBI-Hackathons/svcompare.

## Methods

### Implementation

***SURVIVOR_ant.*** To enable annotation and comparison of the SV callsets, we implemented a new extension of SURVIVOR that aims to assign genomic features, including previously known/established SVs, to merged SV callsets produced by SURVIVOR. SURVIVOR_ant takes any VCF file (list of SVs) as an input (-i) as well as annotation sets specified as a list of BED files (--bed), GFF files (--gff) and additional VCF files (--vcf). Each of the three file types are optional and the user can specify multiple files for each type, separated by commas. SURVIVOR_ant reads in the original VCF file to be annotated and constructs a self-balancing interval tree originally taken from SURVIVOR ( Jeffares *et al.*, 2017). Next, it reads in any annotations in VCF files (e.g. from the 1000 Genomes Project) and compares these to the original VCF entries in the interval tree. The comparison is based on the individual breakpoints, given a maximum distance parameter (by default 1kb). Subsequently, SURVIVOR_ant runs through the BED files and GFF files and parses the provided intervals and identifiers. In the case of a GFF file, SURVIVOR parses the first name in the 9th column: gene=. For BED files, SURVIVOR_ant uses the fourth column as the name for each entry (or the file name, if the BED file does not include a four columns).

**Figure 1.**
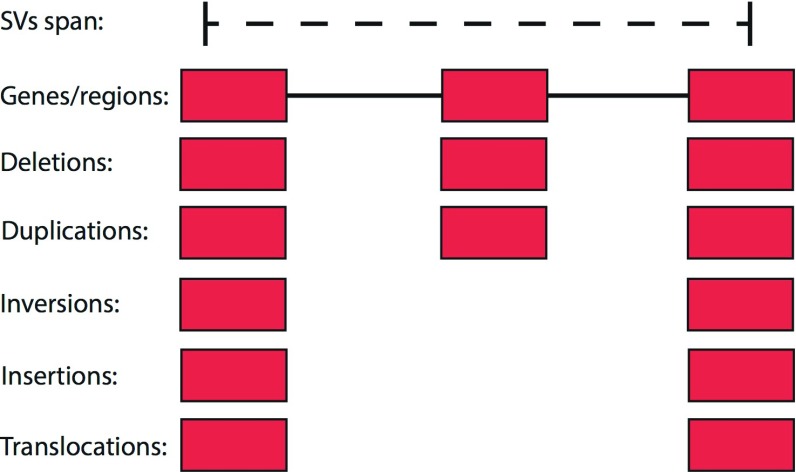
SV type specific overlap schema of SURVIVOR_ant to identify which genomic annotations overlaps with which type of SV. By default SURVIVOR_ant takes 1kbp surrounding the start and stop coordinates into account. Furthermore, for deletions and duplications we take the overlapping regions into account.

Each entry of a BED or GFF file is assigned to deletions and duplications in the SURVIVOR_ant VCF if they overlap the SV +/- a user-defined distance/wobble parameter (by default 1kb). For translocations, insertions and inversions, SURVIVOR_ant only takes the breakpoints into account and assigns genomic features within a user-defined distance/wobble parameter (by default 1kb). Figure 1 shows the schematic based on three genes. The distance/wobble parameter is necessary to account for differences in accuracy of the technology, mapping, or the SV calling algorithm. Often breakpoints are positioned in repeated regions which makes it hard to place the breakpoints accurately.

After all files are read in and compared to the original VCF file, SURVIVOR_ant prints the original VCF file and extends the INFO field with information on how many VCF files have supportive information (“overlapped_VCF=”) as well as how many genomic features within the VCF files could be assigned per original SV (“total_Annotations=”). If SURVIVOR_ant found overlapping genomic features the names associated to these are printed out in a comma separated list (“overlapped_Annotations=”). SURVIVOR_ant is maintained at https://github.com/NCBI-Hackathons/svcompare.

***Summary statistics scripts.*** These statistics were generated with code available at https://github.com/NCBI-Hackathons/svcompare, which analyzes the data as structured by data_structures.pl. The R script stats_plots_v2.R can be used to generate a variety of figures like those in this paper.

***SV analyses.*** Several statistics were computed by event, by call set, and by variant type:

1. The number of callers supporting an event is the count of the number of callers for which SURVIVOR identified a variant call at the same location for an event, subject to the 1 kb wobble parameter and independent of the variant type.
2. For the total number of variant calls per callset by variant type, the SVs of each type were counted separately. For events in which a single caller made more than one call (sub-calls), each sub-call is counted separately (e.g., if a callset has two different deletions that SURVIVOR merged into a single VCF row, it is counted as two deletions).
3. For analysis of the SURVIVOR_ant annotations, the number of events with at least one annotation of the selected type were counted.
4. Breakpoints were compared for events supported by at least four callers, and for which all calls were of the same type. The type was examined for all calls supporting that event, including multiple calls from any one caller. First, the median start and end positions for were calculated for all calls for that event. Second, the distance of each call’s start from the median start, and each call’s end from the median end were computed. For calls with multiple sub-calls from a single caller, the minimum start position and maximum end position were used as the start and end, respectively.

### Operation

SURVIVOR_ant is based on C++ and does not require any preinstalled packages or libraries. The analysis scripts are using BioPerl ( http://bioperl.org/).

***Genomic data.*** We used 16 candidate-SV callsets from the GiaB Ashkenazi son data set available at https://github.com/genome-in-a-bottle. We reformatted the files if they did not correspond to the VCF 4.1 standard then merged the SVs in the 16 files into one multi-sample VCF using SURVIVOR ( Jeffares *et al.*, 2017) with 1 kb as the distance parameter and without requiring type specificity. Next we downloaded three BED files defining repetitive regions from the GA4GH Benchmarking Team at https://github.com/ga4gh/benchmarking-tools/tree/master/resources/stratification-bed-files. Furthermore, we downloaded the gene annotations for GRCh37 from ensembl. Population genomic data was downloaded for the 1000 Genomes Project from dbVar (estd219) ( Sudmant *et al.*, 2015) and filtered to produce a unique set of variant sites. SURVIVOR_ant (Version 0.0.1) was used to annotate the merged SVs with all the annotation data sets. The merged SVs are referred to as “events.”

## Results

We merged the output of 16 different callers containing variants >19 bp ( Table 1) that were run on the Ashkenazi son data (GiaB) using SURVIVOR ( Jeffares *et al.*, 2017). The resulting VCF file contained 134,528 SV events and was annotated by our novel method SURVIVOR_ant. We annotated the SVs with genes from hg19 (GFF), the 1000 Genomes project (dbVar) population-based structural variant calls, and repetitive regions from the GA4GH Benchmarking Team (3 bed files). SURVIVOR_ant compared the five files to the merged SV calls for the Ashkenazi son data within 22 seconds. It identified 4,506 overlapping SVs between the 1000 Genomes Project and our data set ( Table 2). Furthermore, SURVIVOR_ant identified genomic features in the 3 BED files and the GFF gene list overlapping 66,166 SVs out of the total merged 134,528 VCF entries.

**Table 1.** Structural variant callsets for the Ashkenazi son.

Sequencing Technology	Structural Variant Caller	Call Set Name	Reference
Illumina	Mobile Element Insertion finder - no current name	HG002.TE_insertions.recover_filt_mod	( Hénaff *et al.*, 2015)
Illumina	CommonLaw	HG002.commonlaw.deletions.bilkentuniv.082815	( Zhao *et al.*, 2013)
Illumina	FermiKit	HG002.fermikit.sv	( Li, 2015)
Illumina	FreeBayes	HG002_ALLCHROM_hs37d5_novoalign_ Ilmn150bp300X_FB_delgt19	( Garrison & Marth, 2012)
Illumina	GATK Haplotype Caller	HG002_ALLCHROM_hs37d5_novoalign_ Ilmn150bp300X_GATKHC_delgt19	( McKenna *et al.*, 2010)
Illumina	CNVnator	HG002_CNVnator_deletions.hs37d5.sort	( Abyzov *et al.*, 2011)
Illumina	MetaSV	MetaSV_151207_variants	( Mohiyuddin *et al.*, 2015)
PacBio	Assemblytics	hg002.Assemblytics_structural_variants	( Nattestad & Schatz, 2016)
PacBio	MultibreakSV	hg002_attempt1.1_MultibreakSV_mod	( Ritz *et al.*, 2014)
PacBio	Parliament - forced Illumina assembly	parliament.assembly.H002	( English *et al.*, 2015)
PacBio	Parliament - forced PacBio call	parliament.pacbio.H002	( English *et al.*, 2015)
PacBio	smrt-sv	smrt-sv.dip_indel	( Chaisson *et al.*, 2015)
PacBio	Assemblytics	trio2.Assemblytics_structural_variants	( Nattestad & Schatz, 2016)
PacBio	PBHoney	PBHoney_15.8.24_HG002.tails_20	( English *et al.*, 2014)
Complete Genomics	Complete Genomics	vcfBeta-GS000037263-ASM_delgt19	( Carnevali *et al.*, 2012)
Bionano		son_hap_refsplit20160129_1kb	( Mak *et al.*, 2016)

**Table 2.** Summary over the overlapping annotation for the SVs data set.

Annotation type	# of overlapping SVs
Ensembl genes	22,184
Repeats	7,264
1000 genomes SVs	4,506

**Figure 2.**
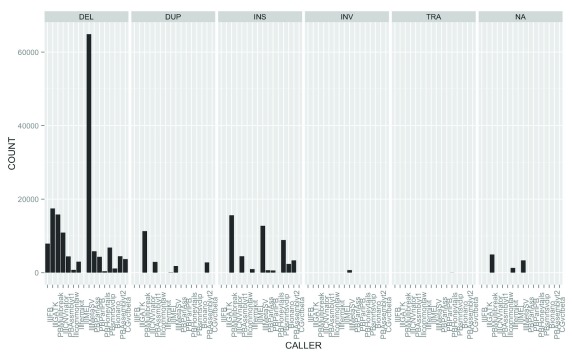
Number of calls per callset for each type of SV, including filtered calls.

The SURVIVOR_ant output is also useful for comparing the output of callers. Each caller assigns an SV type (e.g., insertion, deletion, translocation, etc.) and breakpoints for each SV call. Overall we identified 125,909 (93.6%) SVs that were supported by fewer than four callsets. Figure 2 depicts the widely varying number of candidate SV calls of different types across callsets, which contributes to the large fraction of calls that are supported by fewer than four callsets. Of 134,528 calls from the Ashkenazi son data from the callers in Table 1, 11,474 (8.5%) had more than one SV type discovered by different callers in the same region, and 6,280 (4.7%) had more than one SV type discovered by the same caller in the same region. It is possible either that these different types are due to errors in the calls or that there is a true complex SV consisting of multiple nearby SV types. In addition, duplications of a large region in tandem could be described as an insertion by some callers and as a duplication by other callers. These results illustrate the disagreement of multiple callers over the same data set, as well as the complexity of integrating calls from different methods.

**Figure 3.**
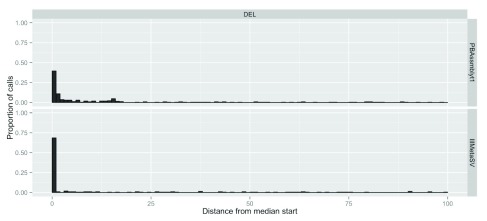
Histogram of distance from the median start position for deletion calls 400 to 999 bp in size for a PacBio-based Assemblytics callset and for an Illumina-based MetaSV callset. Only sites with calls from at least 4 different callsets were included in order to calculate a useful median value at each site.

For characterization of the consistency of breakpoint prediction of the different callers, we analyzed the 5,386 SV events with support from at least four callsets, and for which all calls are of the same type, so that a useful median start and end position could be calculated. Figure 3 depicts example histograms of distance to the median start position for two callsets, one from long reads and one from short reads. In general, more of the short read caller’s start positions are closer to the median breakpoint, but this could be due to a variety of factors, including lower error rates in short reads, easier less repetitive sites detected by short reads, filtering rules, etc. Note that since the number of callers per technology varies and calls supported by more callsets are likely to be easier to detect, this likely introduces a bias in the variants assessed. We calculated these statistics as an example of using our methods, not as a generalizable estimate of breakpoint accuracy.

## Conclusions and future work

In this paper, we introduced SURVIVOR_ant, an annotation and comparison tool especially designed for comparing SVs and genomic features (e.g. genes). SURVIVOR_ant is novel in that it enables a type-specific comparison to multiple genomic annotations and other features of interest. The resulting VCF file can be loaded in existing methods such as IGV or bedtools for further manual inspections. SURVIVOR_ant and all resources used here are available at https://github.com/NCBI-Hackathons/svcompare. This tool is an important first step to enable the comparison of SVs to each other, to known SVs, and to genomic features. Here, we defined genomic features as being information about the properties of the underlying genome sequence (e.g., repetitive regions), as well as annotations such as genes or even chromatin assays. Furthermore, we have made available scripts to calculate a variety of statistics that characterize the similarity and differences between many callsets from a single genome, including the number of callsets supporting similar calls in a region and concordance between their breakpoints.

Future work will include estimating the underlying breakpoints for each SV, potentially based on machine learning methods that utilize information gained from the GiaB consortium on the accuracy of different technologies for different SVs types and sizes. In addition, future work will involve comparing predicted SVs in repetitive regions, since these can often be represented in multiple ways in multiple locations in the genome.

In summary, we present a method (SURVIVOR_ant) for fast annotation of SVs and represents a first step in understanding type and breakpoint concordance for any type of SV, as well as the potential impact of SVs on genes.

## Data and software availability

All the datasets used in this study are available at https://github.com/NCBI-Hackathons/svcompare.gi. Additional raw data can be obtained at https://github.com/genome-in-a-bottle.

Archived source code of the software used as at the time of publication is available at: http://doi.org/10.5281/zenodo.898078 ( dbodian *et al.*, 2017)
