# Supplementary material for: Tools for annotation and comparison of structural variation
Source: F1000Res. 2017 Oct 3;6:1795. [Version 1] doi: 10.12688/f1000research.12516.1 (PMC5668921; doi:10.12688/f1000research.12516.1)
Supplement: Peer review history

# Peer review: Referee response for version 1

**DOI:** 10.5256/f1000research.13552.r26610

**Published:** 2017-11-01

**Authors:** Aaron R. Quinlan

**Affiliations:** 1 Departments of Human Genetics and Biomedical Informatics, USTAR (Utah Science Technology and Research) Center for Genetic Discovery, University of Utah, Salt Lake City, UT, USA

Detecting variation in genome structure is notoriously difficult with existing sequencing technologies and algorithms. Consequently, researchers typically utilize multiple SV detection algorithms, and in some cases, multiple sequencing technologies to maximize accuracy.

 Sedlazeck and colleagues introduce SURVIVOR_ant as a new tool in the SURVIVOR package for annotating a SV callset in VCF format with SV predictions from other tools as well as genome features in BED or GFF format. While the motivation, performance and implementation are sound, I think the authors need to compare their software to both bedtools intersect and especially vcfanno given that vcfanno already provides all or nearly all of the functionality described here. In particular, vcfanno was designed to properly annotate SV events, as it takes the confidence interval about an SV's predicted breakpoints into account. I suggest that the authors extend the manuscript to provide a paragraph comparing the functionality and speed of SURVIVOR_ant to that of vcfanno and bedtools intersect, as this will help readers to better understand its strengths and weaknesses with respect to existing solutions.

 Secondly, I think the authors should state up front exactly what types of events they consider to be SVs, as some call sets listed in Table one restricted to SNPs and short INDELs, which are typically driven by different mutational mechanisms and typically not referred to as SVs.

I have read this submission. I believe that I have an appropriate level of expertise to confirm that it is of an acceptable scientific standard, however I have significant reservations, as outlined above.

## References

[1] PedersenBSLayerRMQuinlanAR: Vcfanno: fast, flexible annotation of genetic variants. *Genome Biol*.2016;17(1) : 10.1186/s13059-016-0973-5 118 10.1186/s13059-016-0973-5 27250555PMC4888505

# Peer review: Referee response for version 1

**DOI:** 10.5256/f1000research.13552.r26611

**Published:** 2017-10-16

**Authors:** Andrew Carroll

**Affiliations:** 1 DNAnexus, Mountain View, CA, USA

This work deals with the methods to compare and overlap structural variant events and presents an analysis of these methods against one of the best studied single genome for structural variant calling - the Genome in a Bottle HG002 - and the extensive population-level structural variant callset from the 1000 Genomes Project. Although in SNP and Indels the problem of determining whether two representations of a call are the same event, this problem is significantly more difficult more structural variant events. In the current state of the field, this is an open and challenging problem.

 The presented analyses are valid. Any review of this work can point out an almost infinite number of parameters for the analysis that can be argued over, such as:

 What are reasonable size ranges to merge events?

 What are proper criteria for filtering events?

 Is it appropriate to include calls from so many methods in merging - should we identify some as unreliable?

 Should certain callers be weighted more highly than other based on reliability?

 How do we consider orthogonal support from different methods - are 3 Illumina methods based on different signals (coverage, insert size) as good as 2 Pacbio?

 Are the differences in how we discover events types (INS/TRA/DEL) significant enough that we need new comparison methods for some?

 What are we missing, but we're not aware we're missing. Do the current methods have any major blindspots?

 Etc... This list could be nearly infinite, mostly because so many questions in this field are unsettled.

 The most important contribution of this work is as a foundation that frames these discussion points. The ability to point to data around a strategy for overlapping and analysis is an important step in progressing beyond discussion of all the items that can be considered.

 The results may be valuable to these tool developers to identify structural variant types, sizes, and genomic contexts which their methods perform poorly on. In addition, the annotation ability may be useful in the annotation and interpretation of structural variant events (one could imagine making BED files for genome regions identified in ExAC or gnomAD as important for and scanning samples or structural variant events that look unusual or damaging in an individual or which impact genes associated with phenotypes of interest).

 The number of possible future directions for this work is unusually large (the questions listed above are a good start).

 There are a number of decisions about the approach that I disagree with and would do differently, but to determine which of those opinions/approaches are valid would require work to the scope of being one (or many) additional publications. 

 I look forward to community efforts which continue to refine the methods to compare and annotate structural variant calls building on the concepts outlined here.

I have read this submission. I believe that I have an appropriate level of expertise to confirm that it is of an acceptable scientific standard.

# Peer review: Referee response for version 1

**DOI:** 10.5256/f1000research.13552.r26608

**Published:** 2017-10-09

**Authors:** Mitchell A. Bekritsky

**Affiliations:** 1 Illumina Cambridge Ltd., Little Chesterford, UK

In this paper, Sedlazeck et al. describe a tool, SURVIVOR_ant, that uses interval trees to intersect multiple SV sets and annotations to produce a single VCF that can be used to compare SV callsets to each other, to known SV datasets, or to other reference annotations. The use of a 'wobble' parameter described in the manuscript enables them to overlap features that are nearby, but may not directly overlap. This is particularly important because SV representations can be pretty variable, although I wouldn't mind seeing a little more direct evidence from the authors motivating the need for this parameter.

 The authors demonstrate an analysis possible with SURVIVOR_ant on 16 HG002 SV callsets, along with the 1000 Genomes Project Phase 3 SV calls, and several reference annotation datasets. While they demonstrate that their analysis produces interesting results, some of their observations might benefit from a little more analysis to round them out. In addition, one of the more interesting points that is apparent in one of the figures is not mentioned in the text, which might be worth bringing up. While I realize this manuscript is not intended to be a thorough analysis of the HG002 SV callsets, it is a very good opportunity for the authors to give their readers some interesting insights into these datasets, and could also help point SURVIVOR_ant users in the direction of some useful questions they can ask once they've run this tool.

 Although no direct analog to SURVIVOR_ant currently exists, some of its functionality overlaps with bedtools intersect. It seems as though their analysis pipeline might be similar to extending the input SV callset by the wobble parameter on either side, translating it into a BED file, then intersecting it with the other datasets. I think SURVIVOR_ant is distinct enough in its focus on SV in VCFs without further intermediate steps that it's a meaningfully distinct tool.

**Software**

 The code for SURVIVOR_ant is written in C++. Some of it could be organized a little better (for instance, the SVS_Node class was in Parser.h, which I found a little confusing). When I tried to compile the code on my Mac, it did not compile because I didn't have a compiler configured with OpenMPI. While that's not a dealbreaker, I'm not sure why there's an OpenMPI requirement in a program with no indication of any parallelization. Perhaps it's from an external library?

 Another concern that I have is the lack of any testing for the interval tree structure implemented by the authors, especially because it can be a somewhat complex data structure to implement. From the header, it looks like this was written a while ago  and might have been taken from another package, where perhaps there was more testing done. If that's the case, it might still be good to include the tests here so that any new bugs might be uncovered by future users of this tool.  If not, perhaps some inspiration can be taken from bedtools's tests?

**Missing wobble parameter implementation?**

 I was able to clone and compile the code on a different machine and run it without any arguments, so the code seems sound. However, I did not attempt to rerun the authors' analysis since the wobble parameter did not appear to be implemented. With this option missing, I did not believe the software provided in the repo linked to the manuscript would allow me to replicate the analysis described by the authors. Not finding any 'wobble' parameter implementation in the C++ code or in the compiled executable, which seems to be a feature that's emphasized in the manuscript, is my primary concern about this manuscript. When I downloaded the source code as at the time of publication, it appeared that the SURVIVOR_ant directory was actually empty. Perhaps the authors neglected to update the code in the repo they refer to in the text to a later version? 

**Wobble intervals**

 The motivation for the wobble parameter could be backed up by a little more data (although I don't doubt its need). Perhaps one of these two plots might be helpful:

1. The length of an event on one axis and the distance to the nearest overlapping event on the other (0 for when the events overlap) might make it clear that lots of events throughout the SV size spectrum that are nearby do not overlap, which would provide more motivation for this parameter.
2. Similarly, for SV calls that are grouped into a single region by SURVIVOR_ant using the wobble parameter, a plot of the reciprocal overlaps for the variants would show that calls that are grouped in a region typically have no overlap, and therefore the wobble parameter is needed.

Additionally, I wonder if a fixed wobble size (defined as 1 kb in the manuscript) is the right choice. My concerns are motivated by 2 factors:

1. For small events, let's say, on the order of 100 bp, extending the interval by 1kb on either side might be a bit liberal, assuming that the size of the SV isn't grossly underestimated and that the breakpoints are in unique regions. In this case, something that's 1kb away from that event might not be a good overlap candidate.
2. For very large events, on the order of 10s to 100s of kb or more, extending the SV on either side by 1kb might conversely be too conservative. Particularly if breakpoints are uncertain, one could imagine the ends of the event moving by 5kb+.

**Additional SV analyses**

 The analyses proposed by the authors are very interesting, but might perhaps be trivially extended to provide additional useful information. One option that might be especially interesting to see is an extension of their 4th analysis proposal (breakpoint variation) to genotype variation. Namely, if 4+ callers have made calls of the same type, it would be good to know if they all have consistent genotypes (e.g. single or double null at a site where a deletion has been called).

 One thing that was unclear to me from the current list of SV analyses is whether there's any per-locus report of the type diversity. It is obviously discussed later in the results section, so I clearly misunderstood one of the analyses described. My money's on 2 or 3. Perhaps the descriptions of these  analyses could be made clearer?

**SVs overlapping with HG002**

 The authors report that 4,506 1000 Genomes SVs overlap with their merged SV set for HG002 out of a total of 134,528 total events. Some context would be helpful for these 2 numbers. In the case of SNPs, F igure 3 from the 1000 Genomes project ^1^ suggests that if the 1000 Genomes project had comprehensively identified all SVs in their study cohort, HG002 should have a significantly higher overlap with previously characterized common SVs. Instead, the data here suggests that just 3% of the SVs identified in HG002 were previously described in the Phase 3 SV release from the 1000 Genomes Project. This is a bit lower than I would expect. There are, of course, possible explanations for this lower overlap that the authors could explore:

- Variants of any type in HG002 are not generally found in the 1000 Genomes Project
- Either due to the methods used or the sequencing data available, the 1000 Genomes project SV dataset is far from complete, and the authors are describing novel SVs.
- It remains possible that many of the novel SVs described in the merged HG002 SV set are false positives (not SURVIVOR_ant's problem, that's a callset problem)

While the point of this paper isn't to thoroughly analyze extant HG002 callsets, this number is a significant enough departure from expectation that it might be worth addressing.

**Breakpoint prediction consistency**

 In the authors' explanation of why short read caller's start positions are closer to the median breakpoint, they posit that the sites detected by short read callers may be easier and less repetitive. Given that the authors have overlapped their calls with the GA4GH repetitive region BED files, they are in a position to substantiate that claim if it were true, or invalidate if it were false. A breakdown of repetitive overlap status for shared calls, calls unique to short read technologies, and calls unique to long read technologies should be done to dig into this claim a little bit more if the authors are going to make it.

 Additionally, the authors that the number of callers per technology varies, but by my count, there are 7 Illumina callers and 7 PacBio callers. At least within the subset of calls detected by either of these 2 technologies, the number of callers is equal, so perhaps it is not a bias is introduced simply due to the number of callers for these 2 technologies.

**Figures**

 Figure 2 is a little difficult to read -- perhaps log-scale the y-axis and color / sort the bars by platform / technology?

 This plot also makes it apparent that many of the calls in this analysis come from a single dataset and a single variant type -- deletions from MetaSV on Illumina data. This is at least worth noting in the manuscript, and it may be worth providing numbers for the analyses done with and without this dataset since it is such a significant outlier. Again, perhaps not the point of the paper, but maybe something the authors could elaborate on a bit.

**Misc**

 A few small suggested edits as well:

- Page 3, Column 2, Paragraph 1 (end of paragraph): if the BED file does not include a four columns -> if the BED file does not include four columns
- Page 4, Column 1, Paragraph 3: The analysis scripts are using BioPerl -> The analysis scripts use BioPerl
- Page 5, Column 2, Paragraph 1: including lower error rates in short reads, easier less repetitive sites detected by short reads, filtering rules, etc. -> including lower error rates in short reads; easier, less repetitive sites detected by short reads; filtering rules, etc. OR including lower error rates in short reads, easier and less repetitive sites detected by short reads, filtering rules, etc.

I have read this submission. I believe that I have an appropriate level of expertise to confirm that it is of an acceptable scientific standard, however I have significant reservations, as outlined above.

## References

[1] 1000 Genomes Project ConsortiumAbecasisGRAltshulerDAutonABrooksLDDurbinRMGibbsRAHurlesMEMcVeanGA: A map of human genome variation from population-scale sequencing. *Nature*.2010;467(7319) : 10.1038/nature09534 1061-73 10.1038/nature09534 20981092PMC3042601

[2] QuinlanARHallIM: BEDTools: a flexible suite of utilities for comparing genomic features. *Bioinformatics*.2010;26(6) : 10.1093/bioinformatics/btq033 841-2 10.1093/bioinformatics/btq033 20110278PMC2832824
